# Death of Dr. Arnold of Ellicottville

**Published:** 1869-03

**Authors:** 


DEATH OF DR. ARNOLD OF ELLICOTTVILLE.—We regret to announce the death of Dr. Arnold of Ellicottville, Cattaraugus Co., N. Y. He had suffered for some months from albuminuria, but continued to pursue his usual business up to within a few days of his death, when his symptoms became rapidly serious, and increasing in severity soon ended in death. He was a very successful physician and much respected citizen, and his early death will be an irreparable loss not only to his relatives and near friends, but to the whole community. We regret our inability to publish a suitable notice of his life and character, and hope some of his acquaintances will furnish us the necessary data.

